# Analysis of efficacy and cost effectiveness of popular weight loss and fitness programs

**DOI:** 10.1186/1550-2783-10-S1-P4

**Published:** 2013-12-06

**Authors:** R Dalton, C Baetge, B Lockard, K Levers, E Galvan, A Jagim, S Simbo, M Byrd, Y Jung, JM Oliver, M Koozehchian, D Khanna, B Sanchez, JY Kresta, K Horrell, T Leopold, M Cho, S Springer, A Rivera, C Cerda, C Rasmussen, R Kreider

**Affiliations:** 1 Exercise & Sport Nutrition Lab, Texas A&M University, College Station, TX 77843, USA

## Background

Obesity is associated with many negative health outcomes. Diet and exercise has been shown to reduce obesity and various other factors linked to poor health. One of the major concerns is the expense of diet and exercise programs. This study compared the cost effectiveness of four popular weight loss programs and controls in terms of weight loss success and outcomes.

## Methods

127 sedentary women (47±11 yr, 45.8±5% body fat, 35.4±5 kg/m^2^) were randomized to participate in a no diet or exercise control group (C) or the Curves Complete^®^ 90-day Challenge (CC), Weight Watchers^®^ Points Plus (WW), Jenny Craig^®^ (JC), or Nutrisystem^®^ Advance Select™ (NS) weight loss programs for 12-wks. Participants in the diet groups were encouraged to exercise (WW, JC, NS) while those in the CC group participated in a structured circuit-style resistance training (3 d/wk) and walking (3/d wk) program. Program and food cost were calculated for a random sample of 1 week for 10 participants for each group. Food costs were estimated based on determining the cost of purchasing foods described in diet logs reported by the participants. These costs were averaged and applied to each subject for the duration of the study. The cost per day (C 4.7±2.2, CC 6.4±1.6, WW 4.9±1.4, JC 2.2±1.1, NS 1.8±1.1 $/day), was used to calculate an average 90 day food cost (C 422±198, CC 579±147, WW 438±130, JC 200±101, NS 162±103 $/90day). This was added to the program participation costs (C 0, CC 300, WW 120, JC 2,400, NS 900 $/90day) to estimate a total cost (C 422±198, CC 879±147, WW 558±130, JC 2,600±101, NS 1,062±103 $/90day) per program. Measurements were taken for body composition, fitness, and health measures. The changes in these variables were then divided by the overall cost for each program to establish the cost effectiveness for each program. Changes from baseline after 12-wks intervention for weight, waist circumference, hip circumference, bone mineral content, fat mass, fat-free mass, and peak oxygen uptake were analyzed by one-way ANOVA.

## Results

Mean ± SD changes for the measured variables are as follows: weight (C 0.22±6.8, CC -11.4±9.1, WW -9.2±7.7, JC -11.7±8.3, NS -11.3±9.8 lbs), waist (C 0.76±2.7, CC -1.5±2.2, WW -1.5±2.5, JC -1.5±1.5, NS -1.3±2.4 inches), hip circumference (C 0.32±1.3, CC -1.9±1.8, WW -1.1±1.1, JC -2.0±1.7, NS -1.7±1.6 inches), fat mass (C -0.03±2.0, CC -4.2.2±4.0, WW -2.2±2.7, JC -3.5±3.3, NS -2.3±2.5 kg), fat-free mass (C 0.1±2.3, CC -0.6±2.4, WW -1.6±2.1, JC -1.8±2.1, NS -2.4±2.2 kg), body fat percentage (C -0.06±1.7, CC -2.86±3.6, WW -0.79±2.4, JC -1.37±2.4, NS -0.19±1.7 %), peak oxygen uptake (C -2.2±5.5, CC 3.0±2.7, WW 0.3±5.5, JC 0.6±4.6, NS 0.8±1.4 ml/kg/min). Participants in the CC and WW groups tended to experience greater losses in weight (C 0.001±0.016; CC -0.013±0.01; WW -0.016±0.01; JC -0.005±0.003; NS -0.011±0.01 lbs/$, p<0.001), waist circumference (C 0.0018±0.006; CC -0.0017±0.003; WW -0.0027±0.004; JC -0.0006±0.001; NS -0.0012±0.002 inches/$, p<0.001), hip circumference (C 0.0008±0.003; CC -0.0022±0.002; WW -0.0020±0.002; JC -0.0008±0.001; NS -0.0016±0.002 inches/$, p<0.001), fat mass (C -0.08±0.04.8; CC -4.8±4.5; WW -4.0±4.9; JC -1.3±1.3; NS -2.2±2.3 g/$, p<0.001), and body fat percentage(C -0.0001±0.004.8; CC -0.0033±0.004; WW -0.0014±0.004; JC -0.0005±0.0009; NS -0.0002±0.0016 %/$, p<0.005) per dollar spent compared to some other diet and exercise interventions. However, the WW group lost more fat-free mass (C 0.33±5.4; CC -0.72±2.8; WW -2.87±3.7; JC -0.69±0.8; NS -2.3±2.1 g/$, p<0.005) per dollar spent compared to the other groups. All intervention groups improved peak oxygen uptake (C -0.0052±0.013; CC 0.0034±0.003; WW 0.0006±0.010; JC 0.0002±0.002; NS 0.0007±0.001 ml/kg/min/$, p<0.005) per dollar spent compared to the control.

## Conclusion

Results indicate that participation in different diet and exercise programs may have variable effects body composition and fitness. The WW group tended to lose a lot of weight and fat mass per dollar spent, but also lost more fat-free mass resulting in a lower change in body fat percentage. The CC group tended to improve peak oxygen uptake and lose more weight and fat mass while preserving fat-free mass resulting in the greatest change in body fat percentage per dollar spent. This analysis suggests diet plus exercise is more beneficial to health and weight loss than diet alone.

## Funding

Supported by Curves International, Waco, TX, USA

